# Modelling the effects of *Spartina alterniflora* invasion on the landscape succession of Yancheng coastal natural wetlands, China

**DOI:** 10.7717/peerj.10400

**Published:** 2020-11-24

**Authors:** Lingjun Dai, Hongyu Liu, Gang Wang, Cheng Wang, Ziru Guo, Yi Zhou, Yufeng Li

**Affiliations:** 1 School of Geography, Nanjing Normal University, Nanjing, China; 2 Key Laboratory of Virtual Geographic Environment (Nanjing Normal University), Ministry of Education, Nanjing, China; 3 Jiangsu Center for Collaborative Innovation in Geographical Information Resource Development and Application, Nanjing, China; 4 School of Marine Science and Engineering, Nanjing Normal University, Nanjing, China

**Keywords:** Yancheng, Native coastal wetlands, Invasive alien species, *Spartina alterniflora*, Cellular-automaton Markov model, Spatial and temporal changes, Sustainable development

## Abstract

**Background:**

The Yancheng coastal natural wetlands (YCNR) are well-preserved silty tidal flat wetlands in China. Due to the severe invasion of *Spartina alterniflora*, the native ecosystem has undergone great changes. The successful invasion of *S. alterniflora* reduced the biodiversity of the YCNR, changed the structure and function of the local ecosystem, and eventually led to the degradation of the ecosystem and the loss of ecosystem function and service. Fully understanding the impact of an alien species invasion on YCNR succession is an important prerequisite for protecting and restoring the wetlands.

**Methods:**

In this study, remote sensing, GIS technology, and a cellular-automaton Markov model were used to simulate the natural succession process of native ecosystems without being affected by alien species. By comparing the landscape of the YCNR with the model simulation results, we gained a better understanding of how alien species affect native landscape-scale ecosystems.

**Results:**

During the natural succession of the coastal native wetland ecosystem in the YCNR, the pioneer species *S. alterniflora* occupied the mudflats and expanded seaward. The whole area expanded and moved seaward with an average annual movement of 58.23 m. Phragmites australis seemed to dominate the competition with *S. salsa* communities, and the area gradually expanded with an average annual movement of 39.89 m. The invasion of *S. alterniflora* changed the native ecosystem’s spatial succession process, causing the *S. salsa* ecosystem to be stressed by ecosystems on the side of the sea (*S. alterniflora*) and that of land (*P. australis*). The area of the seaward-expanding *P. australis* ecosystem has been declining. Under a reasonable protected area policy, human activities have enhanced the succession rate of the P. australis ecosystem and have had a small impact on the ecological spatial succession of *S. salsa* and *S. alterniflora*.

## Introduction

Coastal wetlands are located in the land-sea transition zone (Gómez, White & Wulder, 2016), which is a marginal area and one of the most valuable ecosystems on Earth’s surface (Barbier et al., 2011; Lotze et al., 2006; Smith, 2003). Coastal wetlands have unique functions that cannot be replaced. They are the most biodiverse and highly productive ecosystems (Barbier et al., 2011). They are also the most sensitive areas of the atmosphere, hydrosphere, pedosphere, and biosphere energy and material exchange (Chmura et al., 2003; Jiang et al., 2015; Lotze et al., 2006). *Spartina alterniflora* was introduced to the Yancheng coastal intertidal zone in 1979 (Xu et al., 1989), and a continuous area of *S. alterniflora* has formed since 2000. By 2017, the width of *S. alterniflora* was 1.88 km, the length was 12.90 km and the area was 3,925 ha. The Yancheng coastal natural wetlands (YCNR) are a wetland type that grows and develops in the muddy intertidal zone. Affected by ocean tides, the natural wetland landscape is mainly composed of *Phragmites australis* marshes, *Suaeda salsa* marshes, and mudflats; it develops from land seaward, and it is parallel to the coast (Ou et al., 2004; Zhou et al., 2009). *S. alterniflora* is the main invasive species in China’s coastal zone, and Yancheng is the most significant area affected by *S. alterniflora* invasion. Invasive species directly or indirectly reduce the biodiversity of an invaded area. The consequences are changes in the structure and function of the ecosystem and eventually the degradation of the native ecosystem, reducing its ecological function. Evidence has shown that *S. alterniflora* invasion can have serious ecological consequences by changing the terrain of the intertidal zone (Daehler & Strong, 1996; Gleason et al., 1979), hindering the flow of tidal ditches and water channels, replacing indigenous plants (Callaway & Josselyn, 1992; Chen et al., 2004), crossing with indigenous plants of the same genus, causing the loss of indigenous-plant genotypes or producing hybrids that are more invasive than their parents (Anttila et al., 1998; Ayres et al., 1999), reducing the density and species richness of large benthic invertebrates (Da Cunha Lana & Guiss, 1991; Luiting et al., 1997), hindering fish resource use (Cordell et al., 1998), and reducing key habitats for wintering and water-bird reproduction. Invasive *S. alterniflora* species not only cause biodiversity changes and ecosystem imbalances but also have an important impact on natural-wetland landscape succession. How to effectively manage and control invasive species is an issue of concern to governments and scientists around the world.

*Spartina alterniflora,* a perennial salt-tolerant plant, originated from the west coast of the Atlantic and the Gulf of Mexico, and it is the dominant plant in the low salt marsh of the coastal zone (Bortolus et al., 2019; Bortolus, Carlton & Schwindt, 2015; Peterson et al., 2014). *S. alterniflora* plays an important ecological role in the country of origin. It was unintentionally or intentionally introduced to many countries and regions around the world. It has been shown that there are a series of mechanisms that are beneficial to the survival and diffusion of *S. alterniflora*, for example, seeds, rhizomes, and asexual segments used for rapid propagation and diffusion (Daehler & Strong, 1994); additionally, the species is tolerant to a wide range of changes in ecological factors (Landin, 1991) and has ventilating tissue in the body that can utilize nutrients in the soil under low-oxygen conditions and strong interspecific competition ability (Bertness, 1991; Callaway & Josselyn, 1992; Daehler & Strong, 1996). *S. alterniflora* has become one of the most successful invasive plants in the global coastal salt marsh ecosystem and has received extensive attention. Since the introduction of *S. alterniflora* in the 1970s, it has rapidly expanded in coastal zones (Wang et al., 2018), and the stripe pattern of *S. alterniflora*, *S. salsa*, and *P. australis,* in turn, have expanded from the sea to the land (Sun et al., 2009; Yao et al., 2009).

The Aichi biodiversity target 9 and Sustainable Development Goal 15 targets clearly state that to better protect the diversity of local native ecosystems, we need to address problems such as *S. alterniflora* invasion and develop measures to manage species to prevent invasive alien species from exacerbating ecological damage. Current related research reveals the harmful effects of *S. alterniflora* invasion on regional ecological balance and marine economic development from the perspectives of the ecosystem, ecological processes, structures, and functionality (Chung, 2006; Lu & Zhang, 2013; Zhang, Jiang & Wang, 2009). However, research on how *S. alterniflora* invasion affects native-vegetation-landscape succession requires more attention. To reveal the influence of *S. alterniflora* on the landscape succession of native wetland ecosystems, eight remote-sensing imageries were used as the data source, and the cellular-automaton (CA) Markov model was used to solve two scientific problems on the landscape scale. First, how would the natural landscape of the YCNR develop without *Spartina* invasion? Second, how does *S. alterniflora* structurally affect local landscape succession?

These two scientific issues have important practical guidance for a deep understanding of the impact of *S. alterniflora* invasion on the natural wetland landscape in the intertidal zone of Yancheng, reasonable control of *S. alterniflora*, and protection of the native natural landscape system.

## Materials & Methods

### Study area

**Figure 1:**
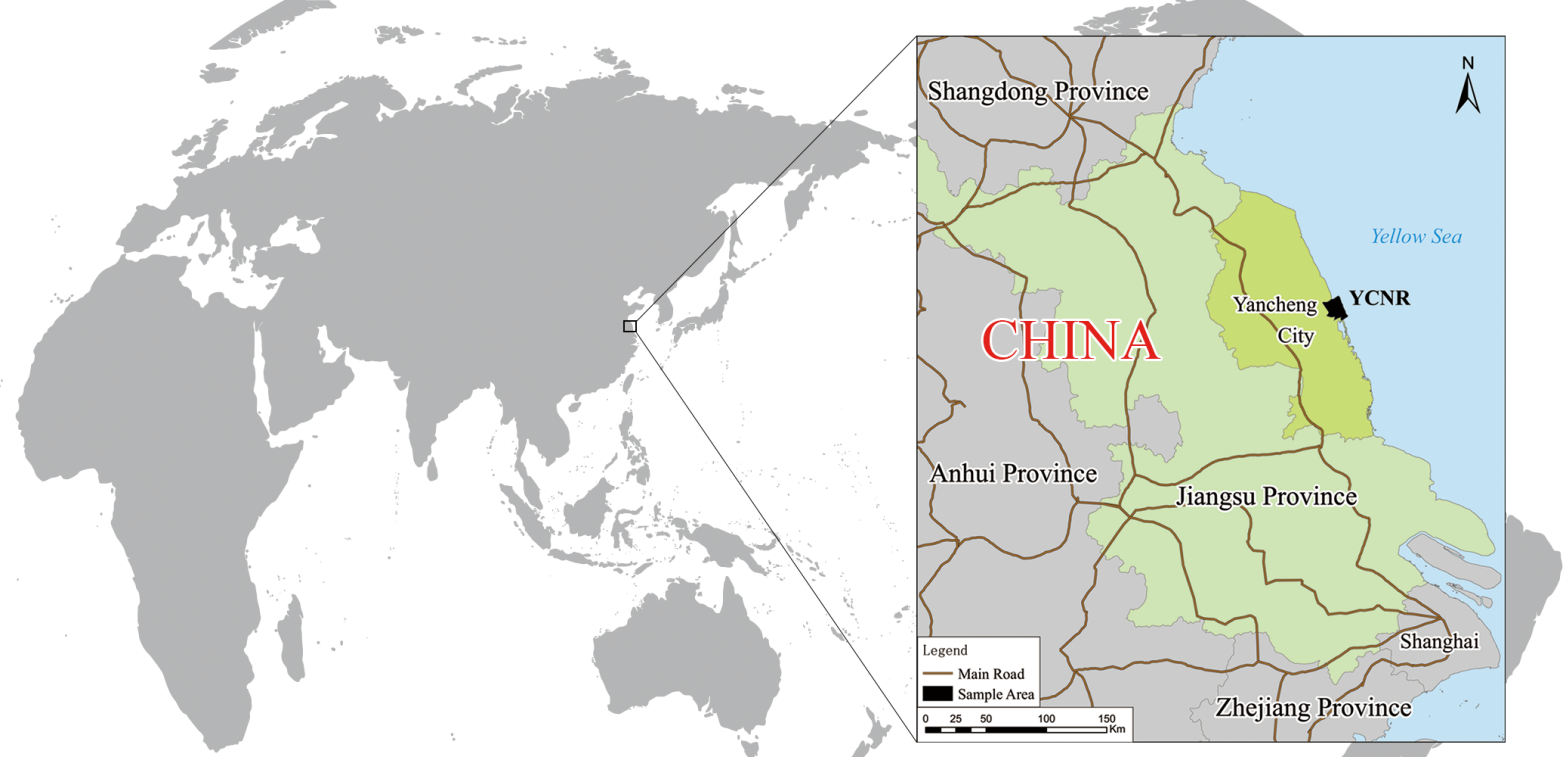
Yancheng coastal natural wetland (YCNR) location in China.

An intertidal zone that is typical of the *S. alterniflora* invasion in Yancheng was selected as the sample area (Fig. 1). This area is the core area of the Yancheng National Rare Birds Nature Reserve in Jiangsu, located at 119°53′45″∼121°18′12″E, 32°48′47″∼34°29′28″N. The reserve mainly protects rare wild animals, such as red-crowned cranes, and their habitat. The reserve is a member of the World Biosphere Reserve Network, a member of the Northeast Asian Crane Protection Network, and a member of the East Asian-Australasian Flyway Partnership. This wetland is one of the important wetlands in the Ramsar Convention and was listed as a World Natural Heritage Site in 2019. The sample area is bounded by the Xinyanggang River to the north and the Doulonggang River to the south. The east is bounded by a 6-m seawater isobath, and the west is bounded by the Sheyang Seawall Road, with an area of 23,830 hectares. The region is in the transition zone of the warm temperate and northern subtropical zones. It is mainly affected by a monsoon climate. The annual average temperature is 13.7–14.6 °C, the annual extreme minimum temperature is −10 °C (January), and the annual extreme maximum temperature is 39.0 °C (August). The frost-free period is 210 to 224 days, and the average annual rainfall is approximately 1,000 mm. The growing season starts in April and ends in November. The soil is marsh coastal saline soil, and the total soil salt is in the range of 0.2%–0.7%. The natural-wetland-landscape system in this area is distributed in strips. Unique soil properties, a suitable climate, hydrological conditions, and an appropriate niche create a very favourable environment and conditions for the survival and spatial expansion of *S. alterniflora*.

### Source and processing of remote-sensing data

To obtain the landscape classification map of the YCNR, we selected these images on the basis of the following criteria: images taken (a) between September and December, when *S. alterniflora* was grown; (b) under appropriate conditions, such as less than 20% cloud cover; and (c) at low tide, so that *S. alterniflora* could be seen growing in the intertidal zone. On the basis of the vector boundary of the study area, a remote-sensing image was cropped in ENVI software and enhanced. The FLAASH atmospheric-correction module was used to apply atmospheric corrections to the remote-sensing image. The FLAASH settings are as follows: (1) coordinates of the scene centre point: it was found from the corresponding HDF file; (2) sensor type: Landsat 5, 7 or 8; (3) altitude: the average altitude of the sample area; (4) flight date and time: it is Greenwich mean time, which can be found in the corresponding HDF file; (5) atmospheric model: mid-latitude winter; (7) aerosol model: rural; and we used the default values for the remaining parameters. The geometric-precision-correction module was used to combine the field control point with the image geometry school. The 8 obtained images were visually interpreted. Water, roads and aquaculture ponds were very easy to distinguish. The NDVI threshold was used when interpreting vegetation. The above raw data were obtained from the International Scientific Data Service Centre (http://www.gscloud.cn). On the basis of remote sensing images from 1985, 1990, 1995, 2000, 2005, 2010, 2015, and 2017 (Table 1), we monitored the dynamic changes in typical coastal wetlands, and the combined transition matrix and CA Markov model were analysed for simulation prediction. The images of 2005 and 2007 were from Landsat 7. We fixed the image bands with the landsat_gapfill patch in ENVI software.

**Table 1:** Remote sensing image metadata information.

**Year**	**Satellite**	**Sensor**	**Acquisition Time**	**WRS Path**	**WRS Row**	**Spatial Resolution**	**Roll Angle**	**Cloud Cover**	**Landsat Scene Identifier**
1985	Landsat 5	TM	24 Sep 1985 01:59:44	119	037	30 m	–	3%	LT51190371985267HAJ00
1990	Landsat 5	TM	8 Oct 1990 01:50:08	119	037	30 m	–	8%	LT51190371990281HAJ00
1995	Landsat 5	TM	9 Dec 1995 01:31:09	119	037	30 m	–	1%	LT51190371995343CLT00
2000	Landsat 7	ETM+	9 Sep 2000 02:21:17	119	037	30 m	–	53%	LE71190372000253EDC01
2005	Landsat 7	ETM+	26 Nov 2005 02:20:07	119	037	30 m	–	6%	LE71190372005330EDC00
2010	Landsat 7	ETM+	10 Dec 2010 02:23:33	119	037	30 m	–	1%	LE71190372010344EDC00
2015	Landsat 8	OLI	13 Oct 2015 02:30:32	119	037	30 m	−0.001	0.04%	LC81190372015286 LGN01
2017	Landsat 8	OLI	5 Dec 2017 02:30:40	119	037	30 m	−0.001	0.58%	LC81190372017339 LGN00

### Model simulation method

The cellular-automaton Markov model method was used for simulation research to reveal the characteristics and regularity of coastal-wetland-landscape succession in the *S. alterniflora* non-invasion scenario. The Markov model is stochastic and mainly used for the simulation of landscape and land-use changes (Adhikari & Southworth, 2012; Baker, 1989; Tine, Perez & Molowny-Horas, 2019; Yu, He & Pan, 2010). Recent studies have shown that the cellular-automaton Markov model is particularly suitable for the simulation of vegetation succession on a landscape scale in areas not affected by human activity (Sun & Liu, 2014). This model uses the previous time step to simulate the next one, and it can therefore be used to simulate the succession trend of a native ecosystem in a state where *S. alterniflora* has not invaded.

Cellular automata and the Markov model are both discrete dynamic models of the temporal state. The cellular automaton model has powerful spatial calculation capabilities, but it is not as good as the Markov model in quantitative calculations. The Markov model mainly focuses on predicting the magnitude of change, but it cannot predict the spatial distribution (Zhang, 2012). Combining the cellular automaton model with the Markov model can construct a model that has both the ability of the CA model to simulate the spatial changes of complex systems and the long-term numerical prediction ability of the Markov model.

The optimal parameters of the simulation effect of this study were as follows: step size: 1 year; filter size: 5 × 5; number of iterations: 20; and cell size: 30 m. We tested the accuracy of the model with a kappa coefficient by using the VALIDATE module in IDRISI Selva software; the kappa coefficient was obtained from statistics on the 1995 interpretation result and 1995 native vegetation landscape maps, which quantitatively reflected the accuracy of the model simulation. Simulation-accuracy parameters were tested: the accuracy rate was 0.9152, and the kappa index exceeded 0.8, reflecting the high credibility of the simulation results and indicating that the CA Markov model could be used to simulate the state of *S. alterniflora* invasion succession of vegetation communities in a typical coastal wetland in Yancheng. More information can be found in Appendix A.

### Mean distance of vegetation-type change

**Figure 2:**
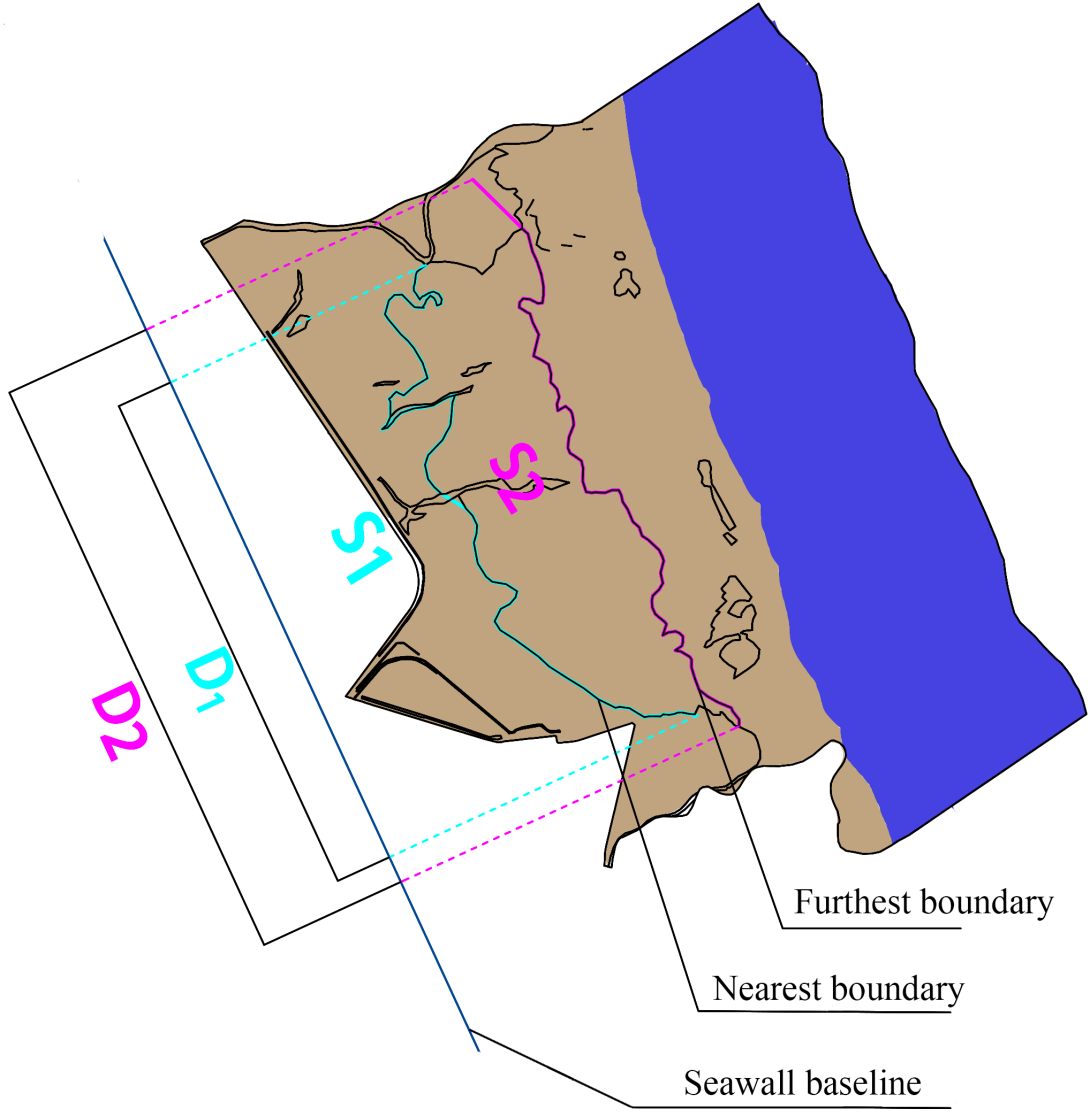
Schematic diagram of mean distance. *S*_1_ is the area enclosed by the nearest boundary and seawall baseline; *S*_2_ is the area enclosed by the farest boundary and seawall baseline; *D*_1_ is the projected length of the nearest boundary on the seawall baseline; *D*_2_ is the projected length of the farthest boundary on the seawall baseline.

The mean distance is used to indicate a trend of vegetation boundary movement. The following formula was used to calculate the mean distance:

(1) \begin{eqnarray*}L=S/D,\end{eqnarray*}

where *L* is the mean distance from the farthest/nearest boundary to the seawall highway; *S* is the seawall highway, which was used as the reference line, perpendicular to the sea and land direction of the seawall highway; the farthest/nearest boundary of the landscape zone and the seawall highway reference line are areas enclosed by vertical lines of the two; and *D* is the projected length of the nearest/farthest boundary on the seawall baseline (Fig. 2).

## Results

### Succession characteristics of a native wetland landscape

The transition probability matrix was obtained by analysing the 1985 and 1990 images (Table 2). The native wetland landscape in the YCNR was obtained by the CA-Markov model. Under native vegetation succession, 87.89% of *S. salsa* still grew in the second year, 11.28% was replaced by *P. australis*, and 0.83% was degraded to mudflat. Then, 98.02% of *P. australis* still grew in the second year, 5.98% was degraded to *S. salsa*, and 0.83% degraded to mudflat.

**Table 2:** Landscape transition probability matrix from 1985 to 1990 (step size is 1 year).

	*S. salsa*	*P. australis*	Mudflat
*S. salsa*	0.8789	0.1128	0.0083
*P. australis*	0.0183	0.9802	0.0015
Mudflat	0.0598	0.0031	0.9370

**Figure 3:**
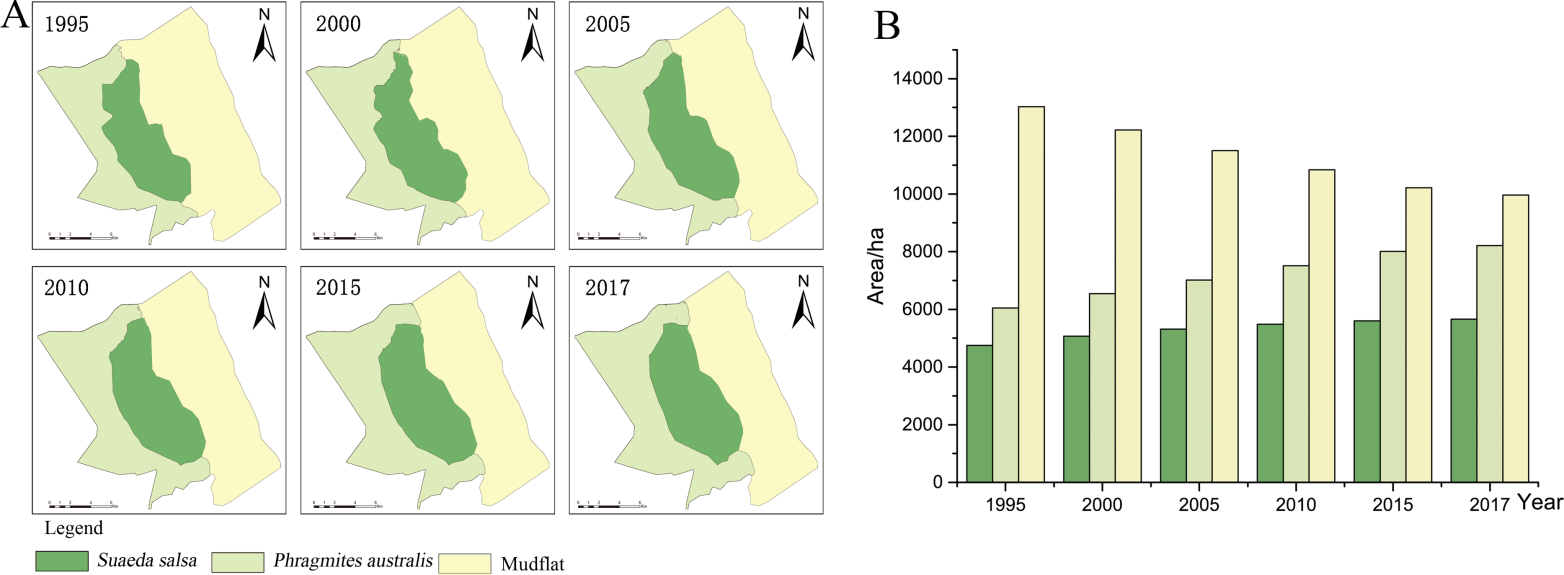
Succession of native vegetation in typical wetlands in YCNR. (A) The simulation results ranged from 1995 to 2017, which is succession of native vegetation in typical wetlands in YCNR. (B) The area statistics of different vegetation.

The cellular automaton Markov model simulated the succession of native landscapes without *S. alterniflora* invasion (Fig. 3). The simulation results showed the succession of native vegetation. The area of native *S. salsa* increased by 909.41 hectares from 1995 to 2017. The area of native *P. australis* continued to increase, with an increase of 2157.21 hectares (35.65%). Community succession occurred at all times in the wetlands. A new *Suaeda* grew on the mudflat, while other *Suaeda* populations were replaced by *P. australis.* From 1990 to 1995, 784.70 hectares of new Suaeda *bonata* grew on the mudflat, while 790.39 hectares of *S. salsa* was replaced by *P. australis*. From 1995 to 2000, the 448.26 hectares of mudflat succeeded in becoming *S. salsa*, and the 534.68 hectares of *S. salsa* turned into *P. australis*. From 2000 to 2005, only 30.71 hectares of mudflat succeeded to *S. salsa*, and 256.58 hectares of *S. salsa* changed to *P. australis*. Between 2010 and 2015, only 46.34 hectares of mudflat area was transformed into *S. salsa,* and 237.11 hectares of *S. salsa* changed into *P. australis*.

With a full YCNR landscape structure and a low degree of fragmentation, the centroid may be used to represent a good succession landscape. The native vegetation distribution was roughly strip-shaped with a complete landscape structure and low landscape fragmentation degree. From the land seaward, the vegetation transitioned from *P. australis* to *S. salsa* to mudflats. Regarding the change direction (Table 3), *P. australis* subsided seaward each year. From 1995 to 2017, the centroid moved 877.50 m seaward, and the average seaward speed was 39.89 m/year. The centroid of *S. salsa* moved seaward. From 1995 to 2017, it moved 1281.10 m seaward with an average speed of 58.23 m/year.

**Figure 4:**
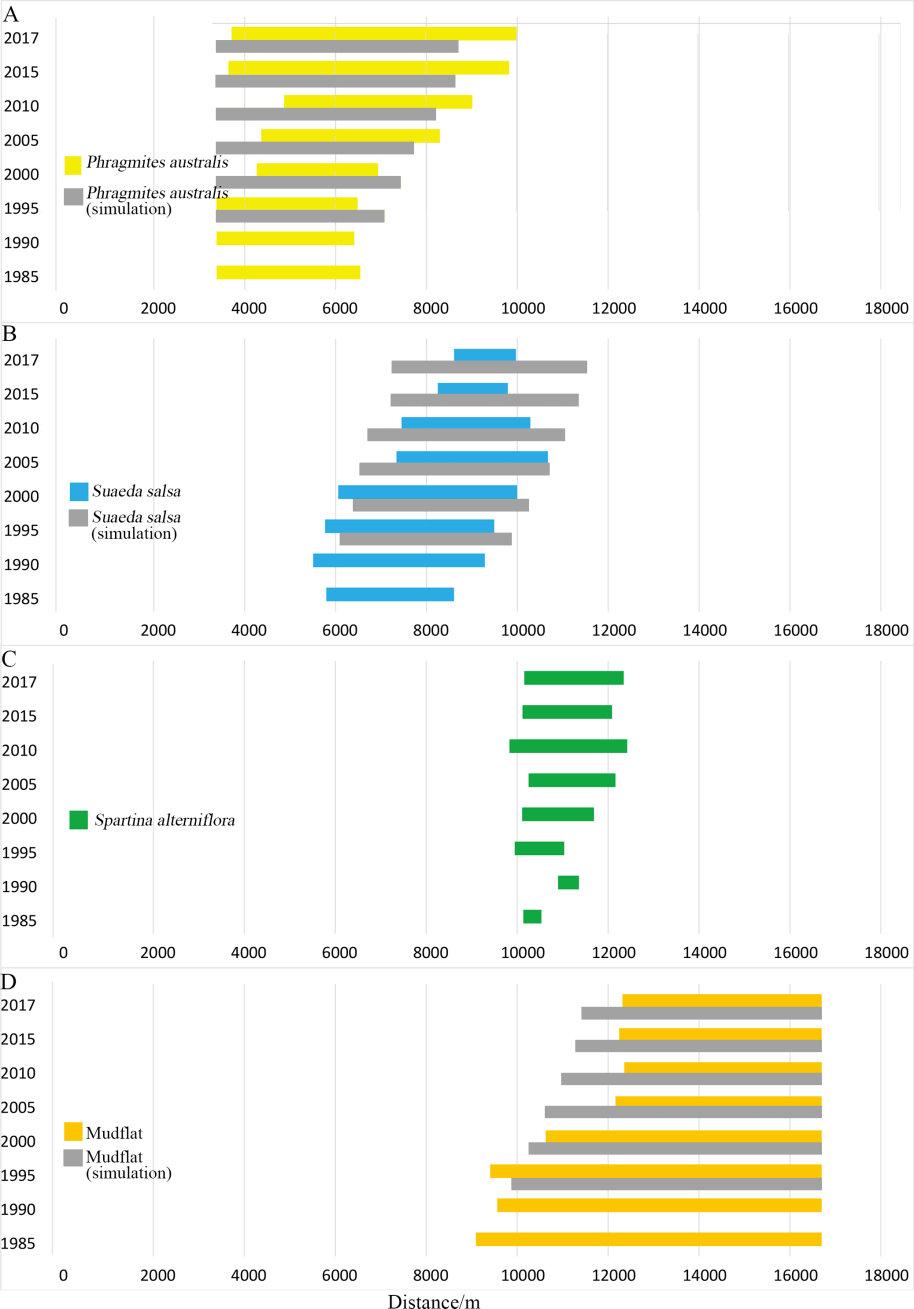
Width distribution of four main landscape types. Detailed data in Table A1. (A) Width distribution of *P. australis*; (B) distribution of *S. salsa*; (C) width distribution of *S. alterniflora*; (D) width distribution of mudflat.

Simulation results (Fig. 4) showed that native *P. australis* was mainly distributed in the range of 3.3–8.8 km from the seawall, and the average patch bandwidth was 4.6 km. Native *S. salsa* was mainly distributed in the range of 6.1–11.5 km from the seawall, and the average patch bandwidth was 4.1 km. The maximum distance of *P. australis* from the seawall baseline moved from the original 14,076.88 m to the land to 11,764.52 m and moved 2323.36 m to the land; the maximum distance from the seawall to the land from the original 12980.36 m moved to the land to 11,816.31 and 1164.05 m.

**Table 3:** Movement of native vegetation centroids.

	**Distance (m)**			**Expansion rate (m/year)**		
	*P. australis*	*S. salsa*	Mudflat	*P. australis*	*S. salsa*	Mudflat
1995	5079.82	7949.92	13243.89			
				37.73	75.81	41.49
2000	5268.48	8328.99	13451.33			
				31.28	67.27	38.69
2005	5424.88	8665.32	13644.77			
				43.32	50.79	34.91
2010	5641.46	8919.29	13819.33			
				45.24	44.48	31.64
2015	5867.64	9141.70	13977.54			
				44.84	44.66	31.91
2017	5957.32	9231.02	14041.36			

### Impact of S. alterniflora invasion on spatiotemporal succession of natural wetland types

#### Spatiotemporal succession of wetland landscape types

Table 4 shows the area of each landscape type in eight periods for a typical coastal wetland. As shown in the table, the area of *S. salsa* first increased and then decreased, the mudflat area decreased, the areas of *S. alterniflora* and *P. australis* increased, and the area of water remained basically unchanged. The area of artificial landscapes represented by aquaculture ponds and roads increased, but this increase was small. Specifically, the area of *S. salsa* increased from 3773.05 hectares in 1985 to 5227.79 hectares in 2000 and then decreased to 992.46 hectares in 2017, with the proportion increasing from 15.83% in 1985 to 21.94% and sharply decreasing to 4.16% in 2017. The area of *P. australis* increased significantly, from 5038.48 hectares in 1985 to 8571.37 hectares in 2017, accounting for an increase ranging from 21.14% to 36.72%, which was 1.74 times the original area. *S. alterniflora* increased from 514.53 hectares in 1985 to 3925.46 hectares in 2017, an increase of 662.92%. The mudflat accounted for more than 35% of the entire landscape ecosystem, and the area decreased from 14,424.51 hectares in 1985 to 8,295.04 hectares in 2017, with the proportion falling from 60.53% to 34.81%. The area of aquaculture ponds maintained an increasing trend from 2000 to 2010. The area of roads increased slowly for 27 years, from 61.00 hectares in 1985 to 1,050.98 hectares in 2017.

**Table 4:** Areas of different landscape types in typical coastal wetlands from 1985 to 2017 (unit: hectare).

	**1985**	**1990**	**1995**	**2000**	**2005**	**2010**	**2015**	**2017**
*S. salsa*	3773.05	4584.83	4771.35	5227.79	3385.65	2656.16	1498.06	992.46
*P. australis*	5038.48	4887.95	4765.92	3221.19	4904.90	5602.39	8242.41	8751.37
*S. alterniflora*	214.53	211.52	680.60	2173.89	3381.34	4318.84	3985.08	3925.46
Mudflat	14,424.51	13,592.65	13,232.36	10,213.61	8640.78	8140.35	8417.34	8295.04
Water	318.86	492.74	328.18	545.36	813.28	445.59	504.19	472.38
Aquaculture ponds				2018.31	2209.76	2261.26	252.81	342.92
Road	61.00	60.82	52.07	430.31	494.83	405.87	930.70	1050.98
Total	23,830.43	23,830.51	23830.47	23,830.46	23,830.54	23,830.46	23,830.60	23,830.60

Typical coastal wetlands in Yancheng have *P. australis*, *S. salsa* and *S. alterniflora* as the main landscape types, and the band distribution was obvious. Viewed from a single landscape (Fig. 4), *P. australis* was mainly distributed on the coast, 3.3–9.9 km from the seawall, with an average width of 4.0 km. From 1985 to 1995, the farthest boundary of *P. australis* continued to move seaward. Over the course of 32 years, it moved 3,443 m seaward at a speed of 107.6 m/year. The width of the *P. australis* landscape belt expanded from 3.16 km in 1985 to 6.28 km in 2017.

The landscape of *S. salsa* was mainly distributed on the coast, 5.8–10.6 km from the seawall baseline, with a mean width of 2.9 km. The nearest boundary of *S. salsa* moved from 5.8 km seaward in 1985 to 8.6 km in 2017, and the farthest boundary of *S. salsa* moved from 8.6 to 9.9 km seaward in 1985, with a movement rate of 42.56 m/year. From 1985 to 2000, the width of the *S. salsa* landscape belt expanded from 2.8 to 3.9 km, with an expansion rate of 75 m/year; from 2000 to 2017, the *S. salsa* belt width decreased sharply to 1.4 km, with a reduction rate of 151 m/year.

The *S. alterniflora* patch was mainly distributed 9.8–12.4 km from the seawall baseline. The mean width of the patch was 1.5 km, and the maximum width was 2.6 km. From 1985 to 1990, *S. alterniflora* was sporadically scattered on the coastal wetlands without banding. From 1985 to 2010, the width of the *S. alterniflora* patch expanded each year from 0.3 to 2.6 km. From 2010 to 2017, the width of the *S. alterniflora* patch decreased from 2.6 to 2.1 km.

#### Impact of *S. alterniflora* invasion on native landscape succession

**Figure 5:**
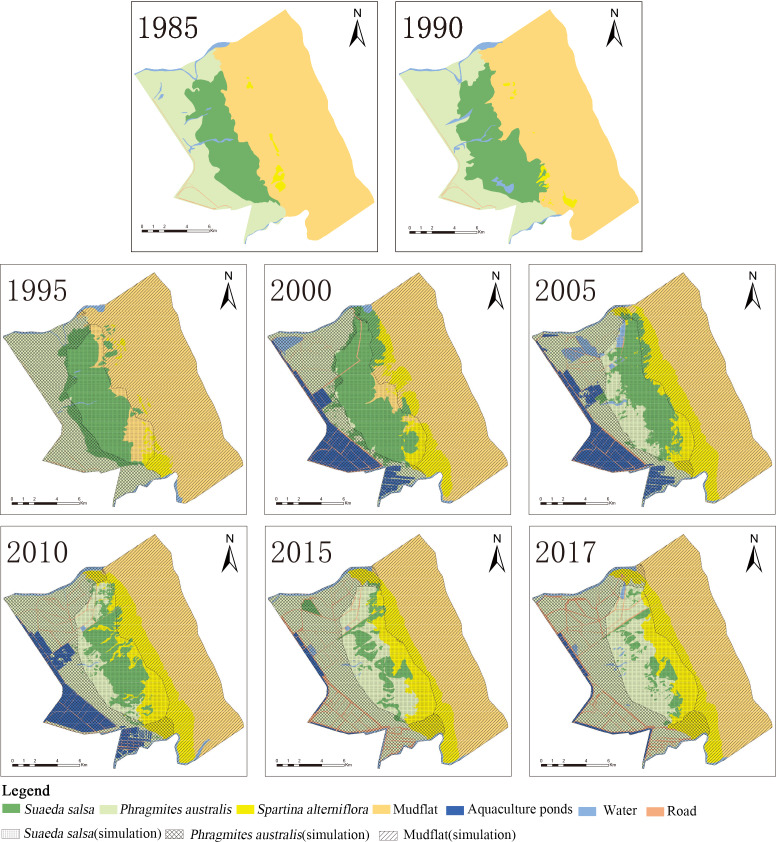
Overlaid map of native vegetation succession and current status. The detailed data are shown in Table A2. (A) The landscape of YCNR in 1985; (B) the landscape of YCNR in 1990; (C) overlaid map of landscape in 1995; (D) overlaid map of landscape in 2000; (E) is overlaid map of landscape in 2005; (F) overlaid map of landscape in 2010; (G) overlaid map of landscape in 2015; (H) overlaid map of landscape in 2017.

*S. alterniflora,* as an alien species in the YCNR, occupies the living space of native species (Fig. 5). A comparison of the simulation and current situation shows that the area of *S. salsa* decreased from 5662.05 to 992.46 hectares of the actual area, and the proportion of the reduced area was 82.47%; the *P. australis*-marsh-wetland area increased from 8208.80 to 8751.37 hectares, accounting for 6.62%. The farthest boundary of *S. salsa* moved from 11,551.51 m to the land to 9997.24 m, and the farthest boundary moved 1581.27 m to the land and moved 1341.18 m seaward. The average landscape bandwidth of *S. salsa* also sharply decreased from 4.3 to 1.4 km. *S. salsa* was forced to contract by receiving stress from both sides, sea and land.

The area of native *S. salsa* was reduced from 3527 hectares in 1995 to 969 hectares in 2017. The area occupied by *P. australis* continued to increase from 5 hectares in 1995 to 3,298 hectares in 2017, accounting for an increase of 0.11% from 53.51%. The area occupied by *S. alterniflora* increased from 167 hectares in 1995 to 1500 hectares in 2017, with an increase of 888.7%. The area occupied by the artificial landscape was less than 100 hectares.

The area of native *P. australis* increased from 4738 hectares in 1995 to 5720 hectares in 2017. The area occupied by *S. alterniflora* continued to increase, accounting for an increase from 0.02% in 1995 to 9.73% in 2017. From 2005 to 2017, the area of native *P. australis* that degraded to *S. salsa* accounted for less than 3%. The area of native *P. australis* that developed into artificial landscapes increased to a maximum of 2610 hectares in 2010 and subsequently decreased to 1284 hectares in 2017.

The simulation results showed that, without being affected by *S. alterniflora*, the farthest/nearest boundaries of the *P. australis* and *S. salsa* landscape patches moved seaward, and the average width of the landscape patch expanded (Table 5). The invasion of *S. alterniflora* affected the structure of the local ecosystem, and the boundary of the near-shore end of *P. australis* was affected by human activities. It moved 1000–1500 m seaward in 2005 and 2010. The farthest *P. australis* boundary moved seaward, but before 2000, it was slower than the simulated succession speed; after 2005, it was faster than the simulated succession speed. The farthest *P. australis* boundary moved seaward faster than the near-land boundary, causing the width of the *P. australis* landscape patch to increase each year. The two borders of the *S. salsa* landscape patch were affected by *P. australis* and *S. alterniflora*. From 1995 to 2017, the nearest boundary of *S. salsa* continued to move seaward. From 1995 to 2000, the nearest boundary of *S. salsa* moved lower than the simulated speed. From 2005 to 2017, the nearest boundary moved seaward increasingly faster than the simulated speed. The speed of the seaward movement of the farthest boundary of the *S. salsa* landscape patch was lower than the simulated speed. The margins of the two ends of the *S. salsa* landscape patch were forced to shrink, resulting in a sharp reduction in the width of the *S. salsa* landscape area.

**Table 5:** Moving distance of landscape boundary (unit: m).

	**Year**	**Landscape Boundary**		**Change in Average Width**
		Nearest Boundary	Farthest Boundary	
*P. australis*	1995	16.09	−598.41	−614.50
	2000	902.61	−507.58	−1410.19
	2005	1001.00	566.82	−434.19
	2010	1503.99	791.54	−712.45
	2015	278.58	1177.33	898.75
	2017	347.04	1278.79	931.74
*S. salsa*	1995	−376.57	−402.07	−25.50
	2000	−355.70	−277.05	78.65
	2005	786.14	−60.76	−846.90
	2010	724.93	−785.47	−1510.39
	2015	1011.10	−1580.20	−2591.30
	2017	1345.18	−1581.27	−2926.45
Mudflat	1995	−461.73	0.75	462.48
	2000	377.86	0.75	−377.12
	2005	1555.81	0.75	−1555.06
	2010	1386.70	0.77	−1385.93
	2015	966.01	0.75	−965.26
	2017	898.39	0.75	−897.64

**Notes.**

A boundary move seaward has a positive value and a move to land has a negative value.

## Discussion

In recent decades, increasing attention has been paid to the study of coastal zones (An et al., 2007; Gao et al., 2014; Hou, Yu & Lu, 2012). China’s marine economy continues to grow steadily, and the scale of development and utilization activities in coastal areas is expanding rapidly (Liu et al., 2015), but the YCNR has preserved the most complete wetland ecosystem, protecting rare wild animals, such as red-crowned cranes, and their habitats (Cao et al., 2016; Jun et al., 2011). The *S. alterniflora* invasion severely changed the natural succession law of the native wetland ecosystem in the intertidal zone of the YCNR (Gao et al., 2014; Hou, Yu & Lu, 2012). The understanding of this issue is limited to ecological studies, and it is difficult to understand the problem of the spatial development of succession stages. Using landscape ecology, with the help of remote sensing and GIS, it is possible to accurately explain the interactions and changes between ecosystems from a larger (landscape) macroscale (Gauch, 1982; Jongman, Braak & Tongeren, 1995; Zhang, 2004).

### The succession of natural wetlands

Seawall roads, rivers, and oceans make the study area a closed, independent system that induces internal and external drivers of system changes. Soil fertility, population competition and other variables are considered internal driving factors, while human activity disturbance and alien species are considered external driving factors. Landscape changes directly reflect the direction and extent of system changes. The CA-Markov model simulated the natural succession of coastal wetlands with only internal drivers. By comparing the model results with the actual landscape structure, we were able to better understand the impact of external drivers on system changes. The succession model of coastal wetland ecosystems was built using a space-for-time substitution approach (Yao et al., 2009). *S. salsa* was the pioneer species, taking the lead in occupying the mudflat. Then, *P. australis* replaced *S. salsa*, and the area of *S. salsa* and *P. australis* continued to increase. This is the law of native vegetation succession. *S. salsa* and *P. australis* populations increased in size and area, and the succession direction moved towards the sea. There were no competing species on the east side of *S. salsa,* and there was sufficient space for growth and nutrition. *S. salsa* spread and grew, but the soil salinity decreased as the north-south direction approached the inland river. The low-salinity soil environment inhibited the growth of *S. salsa*. *P. australis* grew on the west side of *S. salsa*. *P. australis* has more advantages in terms of competing with *S. salsa* for survival space, and *S. salsa* is gradually being replaced by *P. australis*.

External driving factors change the succession of the system. Area change can indicate the specific role of the impact factor. The areas of ponds and roads indicate the impact of human activities on the YCNR. From 2000 to 2010, the proportion of artificial landscapes in the area of native *P. australis* remained at approximately 35% because artificial ponds occupied a large area and kept *P. australis* near the seawall. In 2013, the protected area implemented the policy of “returning fishing and returning wetness” to stop artificial breeding activities, and the artificial breeding ponds were soon restored to reeds. Human activities mainly affected *P. australis* and had less effect on *S. salsa*.

The alien species *S. alterniflora* was introduced into the YCNR, producing hybrids that were more invasive than their parents (Anttila et al., 1998; Ayres et al., 1999; Daehler & Strong, 1997), competing for indigenous plants (Callaway & Josselyn, 1992; Chen et al., 2004; Daehler & Strong, 1996). *S. alterniflora* occupied the living space of native vegetation, suggesting that *S. alterniflora* inhibits the indigenous vegetation, such as common reed and *S. salsa*.

In the natural state, the average grain size of the surface sediment increases from land to sea, and the sorting becomes better (Alexander et al., 1991; Evans, 1965; Ren, 1986). *S. alterniflora* is densely distributed, and its stems and leaves have a strong buffering effect on the flow, which can significantly slow the flow velocity, reduce the tidal current transport capacity, and play a role in capturing suspended sediment, thus increasing the sedimentation rate (Frey & Basan, 1978). The results show that there is a negative feedback between the deposition rate and the elevation of the tidal flat. With the silting up of the tidal flat, the inundation time of tidal water is gradually shortened, and salt-tolerant vegetation such as Artemisia halophyte begins to grow on the beach surface, forming salt marsh wetlands. The elevation of the surface layer of the salt marsh wetland increases due to sediment deposition, which leads to a decrease in the water depth during times of tidal inundation, a reduction in suspended solids brought to the salt marsh, and a shortening of the tidal submergence time, which in turn leads to a decrease in the salt marsh deposition rate (Pethick, 1981).

### Biological dam

**Figure 6:**
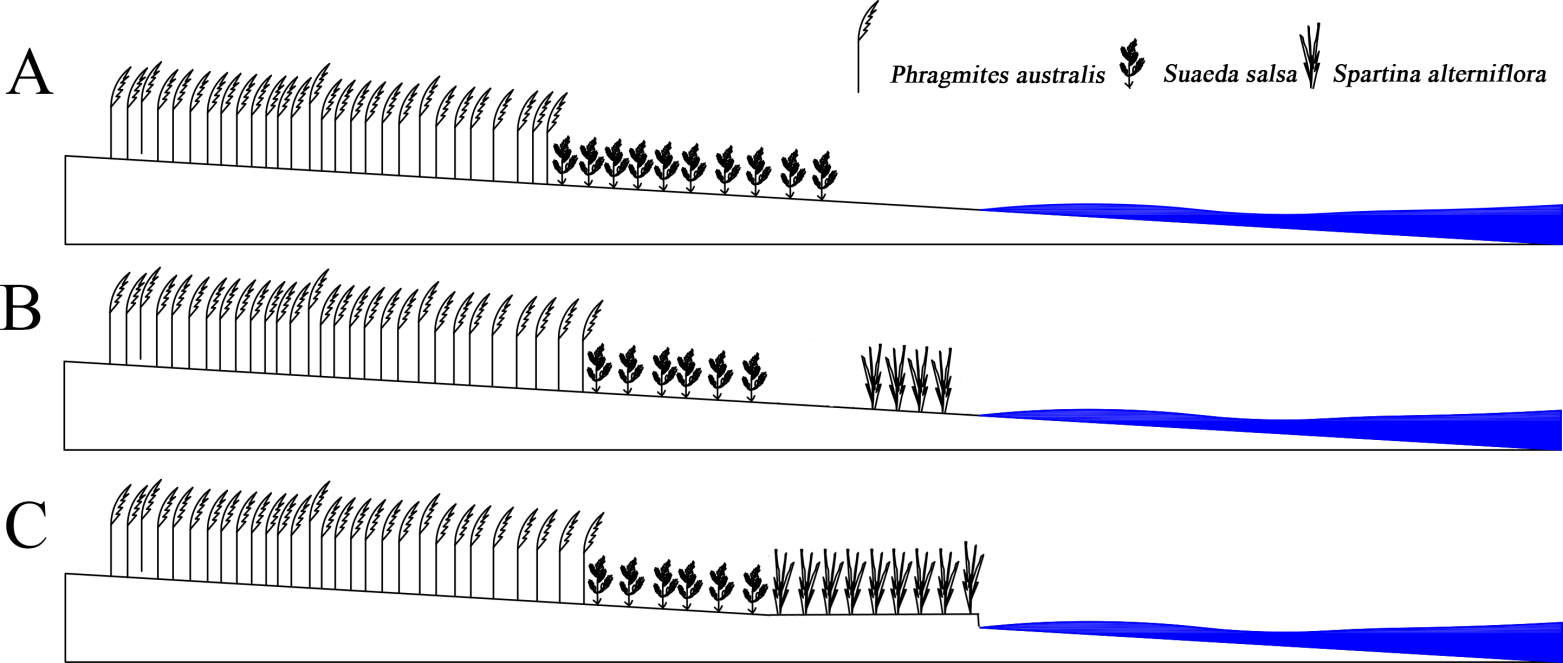
Concept of Biological dam. (A) shows *S. alterniflora* has not yet invaded; (B) shows *S. alterniflora* has just been introduced, and (C) shows that *S. alterniflora* forms a biological dam.

*S. alterniflora* had spread to the entire coastline in just 20 years. After 2005, the area of *S. alterniflora* had not increased significantly, and the width remained unchanged. However, the area of *S. Suaeda* was still rapidly decreasing; the direction of the boundary spread at the far-continent end of *S. salsa* changed from seaward to landward; the speed of the nearest boundary of *S. salsa* moving seaward and was higher than that of the simulated case; the speed of the farthest *P. australis* boundary accelerated towards the sea. The *S. alterniflora* invasion may indirectly alter the internal conditions of the system, and the biological dam conjecture was proposed (Fig. 6). *S. alterniflora* spread into flakes in 2000, and the root system of *S. alterniflora* is thick and dense, which promotes rapid sedimentation and sedimentation (Gleason et al., 1979), changes the terrain of the intertidal zone and prevents the flow of tidal trenches and waterways (Daehler & Strong, 1996). A biological dam was formed in the outermost layer of the coastal wetlands. A biological dam is a hypothetical concept and was composed of *S. alterniflora*. The thick rhizome accelerated sediment deposition, elevated local topography and changed the ecohydrological mechanism (Millard et al., 2013; Zhao-qing et al., 2020). Biological dams weakened the ecological effects of tides on the YCNR. *S. alterniflora* was strip-shaped and weakened the energy of the tidal water movement. Substances carried in seawater were deposited on the mudflats in advance, causing the soil salinity of the *S. alterniflora* zone to increase and reducing the soil salinity of the *S. salsa* zone, inhibiting the succession of *S. salsa* seaward. At the same time, the salt moved downward under the action of precipitation, and under the action of runoff, the salt moved to the sea and was deposited at the biological dam. The reduced soil salinity inside the dam was more suitable for the expansion of *P. australis* populations and inhibited the survival space of *S. salsa*. The biological dam formed by *S. alterniflora* weakened the ability of material exchange between the ocean and wetlands. Wetland soil salinity was reduced, making it more suitable for reed growth. The biological dam conjecture is an ideal model, which needs further study and verification. Climate change and marine hydrological impacts were not considered.

*S. alterniflora* was introduced to promote silt protection. However, it was classified as an invasive species. A sustainable control method is urgently needed to effectively protect the ecosystem and prevent the further invasion of *S. alterniflora.* Effective methods should be studied at the physical, chemical, biological, and ecological levels to inhibit the spread of *S. alterniflora* and eliminate the harm of alien-species invasions that also change native environmental conditions (e.g., geomorphology, hydrology, soil nutrients). It is particularly important to carry out relevant research to restore a suitable living environment for native species. *S. salsa* provides food, water, and habitat for rare birds, such as red-crowned cranes. Artificially expanding *S. salsa* can reduce the harm of species invasion. In addition to knowing the harm posed by a species invasion to an ecosystem, the competition between native species is noteworthy. The *S. alterniflora* invasion might accelerate *P. australis* succession, and it is necessary to understand the driving mechanism to protect *S. salsa* and maintain ecological balance and wetland biodiversity in the tidal flat.

## Conclusions

The *S. alterniflora* invasion is likely to have changed the succession characteristics of the native natural wetland landscape system. According to the CA-Markov model simulation results, the area of *P. australis* and *S. salsa* marsh wetlands increased each year when *S. alterniflora* was not introduced. However, under the influence of *S. alterniflora*, the area of *P. australis* marsh-wetland area slowed its spread, and the area of *S. salsa* marsh wetland decreased each year.

Our results suggest that *S. alterniflora* invasion changed the original natural wetland landscape succession direction. Without being invaded by *S. alterniflora*, the *P. australis* marsh wetland basically did not move in the land section, and the offshore end moved seaward at a speed of 50 m/year; the near-land section of the *S. salsa* marsh wetland moved seaward at a speed of 34.91 m/year. The offshore section moved seaward at a speed of 51.80 m/year. When the *S. alterniflora* marsh wetland had an agglomeration effect in 2005, it forced the *S. salsa* wetland to move toward land and change its succession direction.

*S. alterniflora* and *P. australis* inhibit the development of *S. salsa*. *P. australis* inhibits the expansion of *S. salsa* toward land, and *S. alterniflora* is likely to inhibit the movement of *S. salsa* seaward.

##  Supplemental Information

10.7717/peerj.10400/supp-1 Table S1 Average distance of four main landscape types Unit: m

10.7717/peerj.10400/supp-2 Table S2 Typical wetland simulation and current state transition matrix in Yancheng

10.7717/peerj.10400/supp-3 Data S1 Raw Data Software ArcMap can be used to access this raw data.

## Appendix A

A cellular automaton is a local grid dynamic model of discrete time, space, and state that represents the interaction of space and causality over time. It has powerful spatial computing capabilities and can be used to simulate complex multivariable systems, and the method is suitable for the study of spatiotemporal dynamics of plant communities (Cannas et al., 2004; Li et al., 2007; Wang, Zhang & Guan, 2007). A cellular automaton is composed of the automaton itself, the cell state, the region, and the transformation rules. The basic principle is that the state of the cellular automaton at time T+1 is a function of its state at time T. On the basis of Markov process theory, the Markov dynamic model is used to predict the probability of an event at time T. It can use the transition-probability matrix between states to predict the state and development trend of events. The formula is as follows:

(2) \begin{eqnarray*}S(T+1)=S(T)\times {P}_{ij},\end{eqnarray*}

where S is the system state, T is the time, and Pij is the transition-probability matrix.

This study is based on the cellular automaton-Markov model established by IDRISI software. The kappa coefficient test model was used to simulate accuracy (Bu et al., 2005; Pontius Jr & Schneider, 2001; Pontius, 2001). When the kappa coefficients were 0–0.2, 0.2–0.4, 0.6–0.8, or 0.8–1.0, the reliability of the simulation results was “slide”, “fair”, “model”, “substantial” or “almost perfect”, respectively, calculated as follows:

\begin{eqnarray*}& & Kappa=({P}_{o}-{P}_{c})/(1-{P}_{c}), \end{eqnarray*}

(3) \begin{eqnarray*}& & {P}_{o}=n/N, Pc=1/A,\end{eqnarray*}

where *P*_*o*_ is the correct simulation ratio, *P*_*c*_ is the expected simulation ratio under random conditions, *N* is the total number of grids in the landscape pattern, *n* is the number of correctly simulated grids, and A is the number of land-cover types.

## Additional Information and Declarations

### Data Availability

The following information was supplied regarding data availability: The raw data are available in the Supplemental Files.
