# Supplementary material for: Modelling the effects of Spartina alterniflora invasion on the landscape succession of Yancheng coastal natural wetlands, China
Source: PeerJ. 2020 Nov 24;8:e10400. doi: 10.7717/peerj.10400 (PMC7694568; doi:10.7717/peerj.10400)
Supplement: Table S1 — Unit: m [file peerj-08-10400-s001.docx]

**Table A1.** Average distance of four main landscape types (unit: m).

| ***P. australis*** | **Nearest boundary** | **Patch width** | **Farthest boundary** |
| --- | --- | --- | --- |
| **1985** | **3384.88** | **3162.33** | **6547.21** |
| 1990 | 3383.15 | 3031.11 | 6414.27 |
| 1995 | 3381.40 | 3104.80 | 6486.20 |
| 2000 | 4267.92 | 2669.26 | 6937.18 |
| 2005 | 4366.31 | 3928.21 | 8294.53 |
| 2010 | 4869.30 | 4141.11 | 9010.41 |
| 2015 | 3643.89 | 6177.09 | 9820.98 |
| 2017 | 3712.35 | 6277.81 | 9990.16 |
| ***S. salsa*** | **Nearest boundary** | **Patch width** | **Farthest boundary** |
| 1985 | 5797.61 | 2810.98 | 8608.58 |
| 1990 | 5507.39 | 3777.78 | 9285.16 |
| 1995 | 5771.57 | 3720.36 | 9491.93 |
| 2000 | 6059.88 | 3936.56 | 9996.44 |
| 2005 | 7341.96 | 3330.98 | 10,672.94 |
| 2010 | 7454.31 | 2832.42 | 10,286.74 |
| 2015 | 8251.79 | 1540.49 | 9792.28 |
| 2017 | 8610.46 | 1359.78 | 9970.24 |
| ***S. alterniflora*** | **Nearest boundary** | **Patch width** | **Farthest boundary** |
| 1985 | 10,136.46 | 397.42 | 10,533.88 |
| 1990 | 10,900.96 | 457.25 | 11,358.21 |
| 1995 | 9948.55 | 1087.98 | 11,036.53 |
| 2000 | 10,111.89 | 1578.53 | 11,690.43 |
| 2005 | 10,252.20 | 1910.13 | 12,162.33 |
| 2010 | 9832.03 | 2588.91 | 12,420.95 |
| 2015 | 10,119.01 | 1968.62 | 12,087.63 |
| 2017 | 10,159.34 | 2184.73 | 12,344.07 |
| **Mudflat** | **Nearest boundary** | **Patch width** | **Farthest boundary** |
| 1985 | 9093.70 | 7604.19 | 16,697.89 |
| 1990 | 9564.95 | 7133.06 | 16,698.01 |
| 1995 | 9408.21 | 7289.80 | 16,698.01 |
| 2000 | 10,630.08 | 6067.93 | 16,698.01 |
| 2005 | 12,162.33 | 4535.68 | 16,698.01 |
| 2010 | 12,357.62 | 4340.41 | 16,698.03 |
| 2015 | 12,246.38 | 4451.63 | 16,698.01 |
| 2017 | 12,316.92 | 4381.10 | 16,698.01 |
