# Supplementary material for: Modelling the effects of Spartina alterniflora invasion on the landscape succession of Yancheng coastal natural wetlands, China
Source: PeerJ. 2020 Nov 24;8:e10400. doi: 10.7717/peerj.10400 (PMC7694568; doi:10.7717/peerj.10400)
Supplement: Table S2 [file peerj-08-10400-s002.docx]

**Table A2.** Typical wetland simulation and current state transition matrix in Yancheng.

|  |  | ***S. salsa*** | ***P. australis*** | **Water** | **Mudflat** | ***S. alterniflora*** | **Aquaculture**  **ponds** | | **Roads** | | **Total** | |
| --- | --- | --- | --- | --- | --- | --- | --- | --- | --- | --- | --- | --- |
| 1995 | *S. salsa** | 3527.19 | 5.07 | 22.04 | 1031.45 | 166.89 |  |  | | 4752.64 | |  |
|  |  | 74.22% | 0.11% | 0.46% | 21.70% | 3.51% |  |  |  | 100.00% | |  |
|  | *P. australis** | 1059.08 | 4737.50 | 145.84 | 38.41 | 0.94 |  | 52.07 | | 6033.85 | |  |
|  |  | 17.55% | 78.52% | 2.42% | 0.64% | 0.02% |  | 0.86% | | 100.00% | |  |
|  | Mudflat* | 185.07 | 11.16 | 146.78 | 12150.68 | 512.76 |  |  | | 13006.46 | |  |
|  |  | 1.42% | 0.09% | 1.13% | 93.42% | 3.94% |  |  |  | 100.00% | |  |
|  | Total | 4771.35 | 4753.73 | 314.65 | 13220.55 | 680.60 |  | 52.07 | | 23792.95 | |  |
|  |  | ***S. salsa*** | ***P. australis*** | **Water** | **Mudflat** | ***S. alterniflora*** | **Aquaculture**  **ponds** | **Roads** | | **Total** | |  |
| 2000 | *S. salsa** | 4072.33 | 73.96 | 0.66 | 420.82 | 454.08 |  | 48.69 | | 5070.53 | |  |
|  |  | 80.31% | 1.46% | 0.01% | 8.30% | 8.96% |  | 0.96% | | 100.00% | |  |
|  | *P. australis** | 548.93 | 3143.43 | 417.06 |  | 26.15 | 2017.36 | 371.12 | | 6524.04 | |  |
|  |  | 8.41% | 48.18% | 6.39% |  | 0.40% | 30.92% | 5.69% | | 100.00% | |  |
|  | Mudflat* | 606.53 | 2.62 | 112.98 | 9781.70 | 1693.59 |  | 1.98 | | 12,199.40 | |  |
|  |  | 4.97% | 0.02% | 0.93% | 80.18% | 13.88% |  | 0.02% | | 100.00% | |  |
|  | Total | 5227.79 | 3220.00 | 530.69 | 10,202.51 | 2173.82 | 2017.36 | 421.79 | | 23,793.97 | |  |
|  |  | ***S. salsa*** | ***P. australis*** | **Water** | **Mudflat** | ***S. alterniflora*** | **Aquaculture**  **ponds** | **Roads** | | **Total** | |  |
| 2005 | *S. salsa** | 2936.66 | 1467.96 | 183.67 |  | 694.48 |  | 28.98 | | 5311.75 | |  |
|  |  | 55.29% | 27.64% | 3.46% |  | 13.07% |  | 0.55% | | 100.00% | |  |
|  | *P. australis** | 122.80 | 3435.75 | 547.86 | 9.20 | 215.80 | 2208.80 | 457.33 | | 6997.55 | |  |
|  |  | 1.75% | 49.10% | 7.83% | 0.13% | 3.08% | 31.57% | 6.54% | | 100.00% | |  |
|  | Mudflat* | 326.19 |  | 68.85 | 8618.67 | 2471.06 |  |  | | 11484.77 | |  |
|  |  | 2.84% |  | 0.60% | 75.04% | 21.52% |  |  |  | 100.00% | |  |
|  | Total | 3385.65 | 4903.71 | 800.38 | 8627.88 | 3381.34 | 2208.80 | 486.31 | | 23,794.07 | |  |
|  |  | ***S. salsa*** | ***P. australis*** | **Water** | **Mudflat** | ***S. alterniflora*** | **Aquaculture**  **ponds** | **Roads** | | **Total** | |  |
| 2010 | *S. salsa** | 2491.17 | 1536.04 | 30.78 |  | 1376.95 |  | 45.57 | | 5480.51 | |  |
|  |  | 45.46% | 28.03% | 0.56% |  | 25.12% |  | 0.83% | | 100.00% | |  |
|  | *P. australis** | 74.31 | 4064.35 | 279.47 | 11.98 | 450.91 | 2259.43 | 350.07 | | 7490.52 | |  |
|  |  | 0.99% | 54.26% | 3.73% | 0.16% | 6.02% | 30.16% | 4.67% | | 100.00% | |  |
|  | Mudflat* | 90.69 | 1.34 | 117.85 | 8117.64 | 2490.97 |  | 3.21 | | 10,821.71 | |  |
|  |  | 0.84% | 0.01% | 1.09% | 75.01% | 23.02% |  | 0.03% | | 100.00% | |  |
|  | Total | 2656.16 | 5601.73 | 428.10 | 8129.63 | 4318.84 | 2259.43 | 398.85 | | 23,792.74 | |  |
|  |  | ***S. salsa*** | ***P. australis*** | **Water** | **Mudflat** | ***S. alterniflora*** | **Aquaculture**  **ponds** | **Roads** | | **Total** | |  |
| 2015 | *S. salsa** | 1299.69 | 2631.75 | 29.51 |  | 1577.78 |  | 62.25 | | 5600.98 | |  |
|  |  | 23.20% | 46.99% | 0.53% |  | 28.17% |  | 1.11% | | 100.00% | |  |
|  | *P. australis** | 188.32 | 5610.07 | 398.54 | 19.45 | 662.48 | 247.40 | 862.93 | | 7989.20 | |  |
|  |  | 2.36% | 70.22% | 4.99% | 0.24% | 8.29% | 3.10% | 10.8% | | 100.00% | |  |
|  | Mudflat* | 10.04 |  | 60.10 | 8387.10 | 1744.82 |  | 2.09 | | 10204.16 | |  |
|  |  | 0.10% |  | 0.59% | 82.19% | 17.10% |  | 0.02% | | 100.00% | |  |
|  | Total | 1498.06 | 8241.82 | 488.15 | 8406.55 | 3985.08 | 247.40 | 927.27 | | 23,794.33 | |  |
|  |  | ***S. salsa*** | ***P. australis*** | **Water** | **Mudflat** | ***S. alterniflora*** | **Aquaculture**  **ponds** | **Roads** | | **Total** | |  |
| 2017 | *S. Salsa** | 968.77 | 3029.78 | 68.95 |  | 1499.99 |  | 94.57 | | 5662.05 | |  |
|  |  | 17.11% | 53.51% | 1.22% |  | 26.49% |  | 1.67% | | 100.00% | |  |
|  | *P. australis** | 23.69 | 5719.80 | 332.05 | 57.79 | 766.48 | 339.32 | 944.47 | | 8183.60 | |  |
|  |  | 0.29% | 69.89% | 4.06% | 0.71% | 9.37% | 4.15% | 11.54% | | 100.00% | |  |
|  | Mudflat* |  | 0.91 | 57.44 | 8226.30 | 1658.99 |  | 4.97 | | 9948.61 | |  |
|  |  |  | 0.01% | 0.58% | 82.69% | 16.68% |  | 0.05% | | 100.00% | |  |
|  | Total | 992.46 | 8750.49 | 458.43 | 8284.10 | 3925.46 | 339.32 | 1044.01 | | 23,794.26 | |  |

Note: numerator represents area, denominator represents percentage
